# The Global Research Trends and Hotspots on Developmental Dysplasia of the Hip: A Bibliometric and Visualized Study

**DOI:** 10.3389/fsurg.2021.671403

**Published:** 2021-10-25

**Authors:** Haiyang Wu, Yulin Wang, Linjian Tong, Hua Yan, Zhiming Sun

**Affiliations:** ^1^Graduate School of Tianjin Medical University, Tianjin, China; ^2^Clinical College of Neurology, Neurosurgery and Neurorehabilitation, Tianjin Medical University, Tianjin, China; ^3^Tianjin Key Laboratory of Cerebral Vascular and Neurodegenerative Diseases, Tianjin Neurosurgical Institute, Tianjin Huanhu Hospital, Tianjin, China; ^4^Department of Orthopaedic Surgery, Tianjin Huanhu Hospital, Tianjin, China

**Keywords:** developmental dysplasia of the hip, bibliometric analysis, visualization, research trends, hotspots

## Abstract

**Background:** Developmental dysplasia of the hip (DDH) is a common musculoskeletal disorder in newborns and also one of the most common causes of hip arthritis in women. Many topics concerning DDH still remain controversial, and the global research trend in this field has not been well-studied yet. The aim of the present study was to illustrate the overall knowledge structure, development trends, and research hotspots of DDH.

**Methods:** The publications related to DDH from 1998 to 2020 were identified from the Web of Science Core Collection (WOSCC). Three bibliometric tools were used to conduct visualization and knowledge maps. Annual trends of publications, contributions of countries, institutions, authors, funding agencies and journals, and clustering of keywords were analyzed.

**Results:** A total of 2,691 publications were included. The annual number of DDH publications showed an increasing trend worldwide. The United States has made the greatest contribution, with the largest number of publications and the highest H-index. The most prolific institutions were Shanghai Jiao Tong University, Children's Hospital of Philadelphia, and Shriners Hospital for Children. Professors Tönnis D, Harris WH, Crowe JF, Graf R, and Salter RB have made great achievements in this field. However, the collaboration between international institutions or researchers was relatively low and mainly conducted in European and American countries. All the keywords could be divided into five clusters: hip osteoarthritis study, hip replacement study, hip ultrasound study, osteotomy surgery study, and etiology study. A trend of balanced and diversified development existed in these clusters. Keywords with the ongoing bursts, including clinical outcome, risk factor, femoroacetabular impingement, predictor, arthroscopy, morphology, and anteversion may continue to be the research hotspots in the near future.

**Conclusions:** There will be an increasing number of publications on DDH research, and the United States stay ahead in this field. International collaboration needs to be further strengthened. The information can provide helpful references for researchers to explore hot issues or target a specific field of DDH.

## Introduction

Developmental dysplasia of the hip (DDH) is a congenital, developmental deformation, or misalignment of the hip joint, which is affected by genetic, environmental, and mechanical factors. The definition encompasses a broad range of presentations, from mild acetabular dysplasia, deficient coverage of the femoral head; that the incidence of DDH in newborns ranges from 1 to 7% across several distinct populations results from differing genetic predisposition and cultural practices (1–4). Developmental dysplasia of the hip is one of the most common limb deformities in children and a frequent cause of secondary osteoarthritis. In developed countries, the resultant socioeconomic burden can be up to tens of millions of dollars for screening DDH (5).

**Figure 1:**
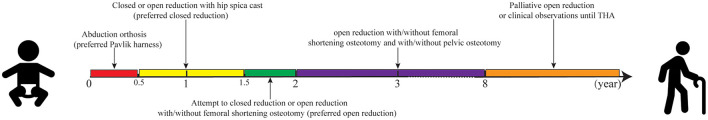
Treatment principles of developmental dysplasia of the hip (DDH) based on age.

The treatment algorithm of DDH mainly depends on the age of patients and the severity of their condition. Although variations in treatment exist based on individual patient characteristics, the general treatment algorithm for DDH was shown in Figure 1. At early stages, if properly treated, the disease can be progressively reversed back to normal (6). From a technical standpoint, the goal in the early treatment is to achieve and maintain the concentric reduction of the acetabulum and femoral head to allow for continuing normal development of the hip. However, if allowed to natural progress without treatment, the femoral head may gradually displace proximally or laterally, leading to accelerated degeneration of the articular cartilage and hip osteoarthritis in young adults (7, 8). Previous studies showed that approximately 17% of patients with DDH requiring the open surgical intervention of a dislocated hip in childhood may eventually progress to total hip arthroplasty (THA). In other cases, DDH is usually asymptomatic until patients developed hip pain or dysfunction associated with degenerative changes (9, 10).

In the last two decades, there have been tremendous advancements in diagnostic and therapeutic strategies of DDH, but many questions still remain unresolved and controversial, such as risk factors in DDH, screening methods, optimal surgical timing, imaging modalities to guide treatment, predictors of a treatment outcome, and so on (11–14). In recent years, as the increasing and deeper studies, an increasing number of papers on the etiology, pathogenesis, diagnosis, and treatment of DDH have been published. However, to the best of our knowledge, the global research trend and hotspots in DDH have not yet been systematically studied. Thus, it is necessary to investigate the overview and current status of the DDH research.

Bibliometric analysis is an interdisciplinary method that combines statistical methods with data visualization technology to identify research frontiers, development trends, and rising patterns of specific subjects. Several commercial visualization tools, such as VOSviewer (15), Citespace (16), Pajek (17), and BibExcel (18), are available nowadays and enable investigators to create knowledge network maps and trace scientific developments. To date, bibliometric analysis has been extensively used in orthopedic fields for estimating the research trends of orthopedic disorders and surgical approaches, such as knee osteoarthritis (19), hip fracture (20), orthopedic oncology (21), atlantoaxial spine surgery, and joint replacement surgery (22, 23). Therefore, this study aimed to use a bibliometric method to intuitively show the overall research framework, development trends, and research hotspots in the field of DDH for the first time. In addition, we hope this research can help scientific researchers to better understand the current status and frontier trends, and continue to deepen the basic and clinical research.

## Methods

### Data Source

Although a variety of databases, such as PubMed, Google Scholar, and Scopus, are able to meet the requirement for evaluating the global DDH research trends, the data for this study were collected from the Science Citation Index Expanded (SCI-Expanded) of Web of Science Core Collection (WOSCC) (19, 24). Web of Science (WOS) is a large-scale, systematic, and multidisciplinary authoritative database, which contains numerous influential high-quality journals from countries worldwide (19, 25). It is also one of the most frequently used databases in previous bibliometrics studies (24, 25).

### Retrieval Strategies

**Figure 2:**
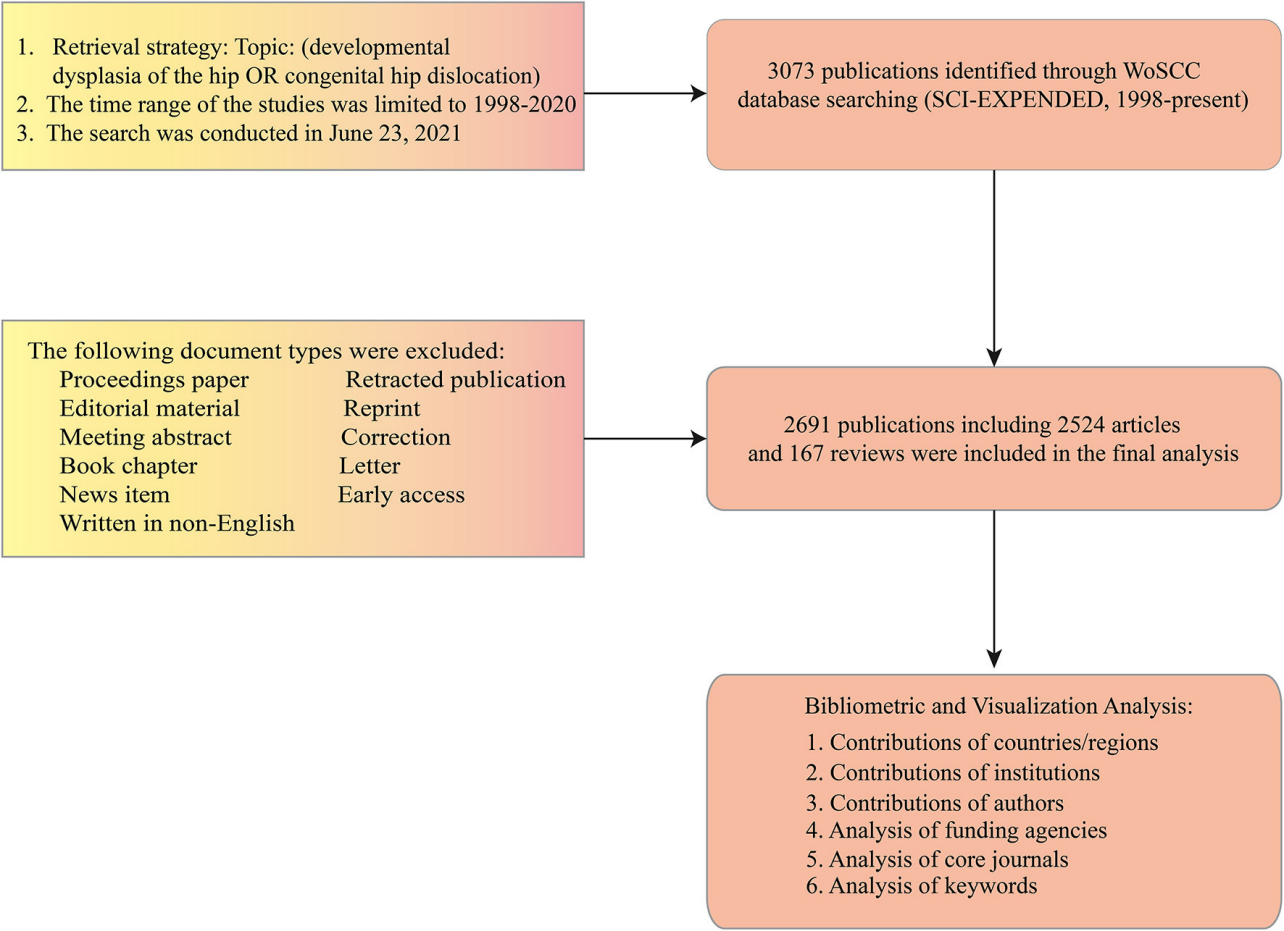
Flowchart for the selection of literature included in the study.

Retrieval work was performed all within 1 day (June 23, 2021) in case of variations brought about by daily updates of the database. The retrieval strategy was as follows: Topic: (developmental dysplasia of the hip) or Topic: (congenital hip dislocation) and Language: (English). The publication types were limited to original articles and reviews, excluding letters, editorials, meeting abstract, and news reports. The time frame of publications was focused on 1998–2020. All records, including the titles, authors, abstracts, keywords, and references, were downloaded and exported in plain text format (Figure 2). The data are all secondary and do not contain any personal identification information. So informed consent statement was not required.

### Data Extraction and Descriptive Analysis

These TXT files were imported into Microsoft Excel 2019 (Microsoft Corporation, Redmond, Washington, USA) for further data processing and graph plotting. Data extracted from the selected articles include the general information about the annual amount of publications, citation frequency, average citation per item (ACI), original countries and institutions, authors, journals, and H-index. Data entry, cleaning, and descriptive statistical analysis were conducted manually in Microsoft Excel. GraphPad Prism 8.0 (GraphPad Software Inc.) was also applied to analyze data and create graphs.

### Bibliometric and Visualized Analysis

VOSviewer (15) [Version 1.6.16, Eck and Waltman (15), the Netherlands], Citespace V (16) [Version 5.7 R5, Chaomei Chen (16), Drexel University, USA], and an online analysis platform were used to perform this bibliometric study. VOSviewer was used to visualizing co-authorship of countries, co-citation of journals, and keyword co-occurrence. In the network visualization map created by using VOSviewer, different nodes indicated various parameters, such as countries, journals, and keywords, while the sizes of the nodes in the map were proportional to the number of publications, citations, or occurrences. Co-authorship or co-citation relationships between the nodes were represented as links. Total link strength (TLS) was used as a weight attribute to indicate the total strength of the links of the selected nodes (19, 20).

CiteSpace was also used to perform cooperation and co-citation analyses of institutions or authors, the dual-map overlay of journals, and burst keywords. In the network map of cooperation or co-citation, each node also represented the type of the element being analyzed, and its size represented the number of publications or citations. Betweenness centrality (BC) is an important parameter of centrality that could assess the scientific importance of the nodes in a network, and nodes with high BC-value (≥0.1) are usually indicated by purple rings (26). In the clusters view map, cited authors with similar categories were gathered in a cluster. All the clusters were labeled in different colors, and the links between nodes represented elements cited together. As for the dual-map overlay of journals, the labels represented different research subjects covered by the relevant journals. The left side of the map was citing journals, while the right side was cited journals. Different colors and widths of lines originating from the citing map and ending at the cited map indicated the paths of the citation links. The path widths were scaled proportionally to the frequency of z-score-scale citation. Keywords with citation bursts refer to those that have been frequently cited for a period, which means that the keywords have received special attention from associated academic researchers.

## Results

### Global Publication and Citation Trend

**Figure 3:**
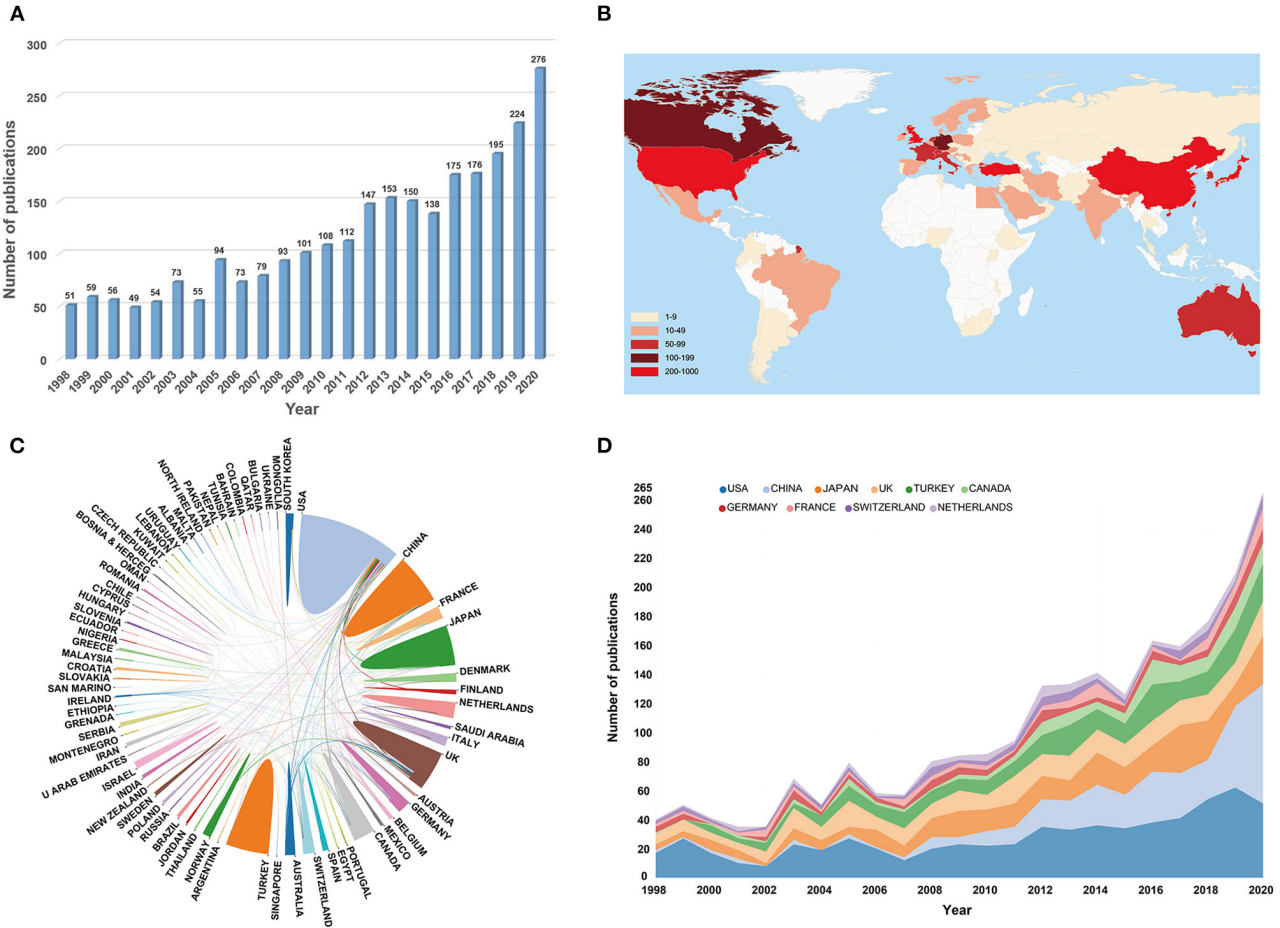
**(A)** The number of annual publications on DDH from 1998 to 2020. **(B)** A world map displaying the global scientific output of DDH research. These different colors in the map represent different density values. **(C)** The cooperation map of countries/regions involved in DDH research. The color area indicates the number of publications. The larger the area, the more the counts. The lines represent the collaborative relationship between countries, and the thicker line indicates a closer relationship. This map was generated from an online analysis platform (https://bibliometric.com). **(D)** The publication trends of the top 10 active countries in DDH research from 1998 to 2020. The width of the line in different colors could reflect the changing trend of annual publications in different countries at different time points.

Based on the selection criteria, a total of 2,691 publications, including 2,524 original articles and 167 reviews, were identified from 1998 to 2020 (Figure 2). As presented in Figure 3A, the trend of global DDH research publications follows an exponential increase in the past 23 years. The number of publications has increased from 51 (1998) to 276 (2020), and almost 38.9% of them (1,046 papers) were published over the last 5 years. Similar to the change of publications, there is also an ascending trend in the number of citations yearly (Supplementary Figure 1).

### Contributions of Countries/Regions

**Figure 4:**
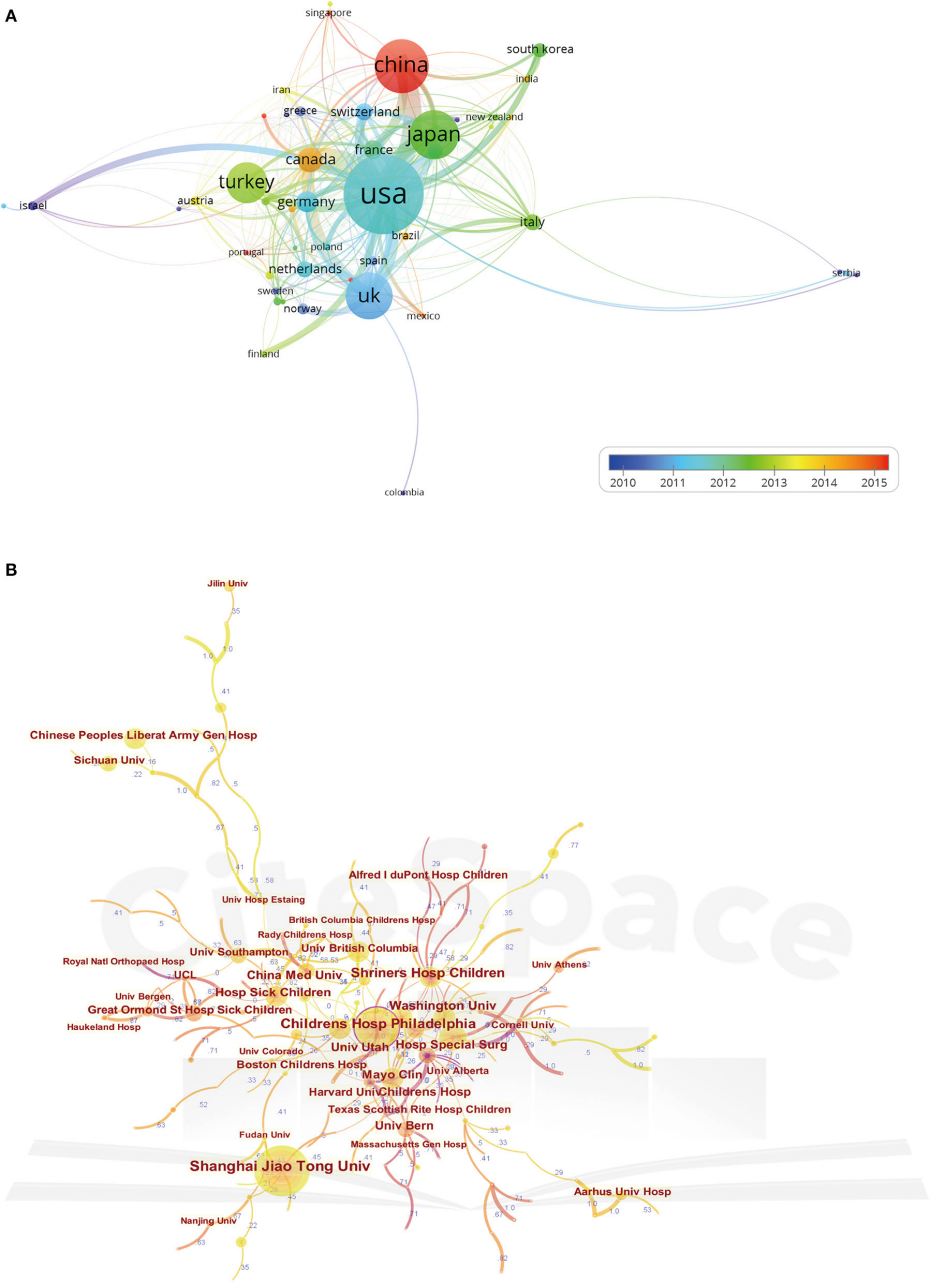
**(A)** The co-authorship map of countries/regions involved in DDH research (generated by VOSviewer). Each node represents a different country, and the node size is proportionate to the number of publications. Different nodes were coded with different colors, depending on the average appearing year (AAY) of the country. Specific division information can be seen in the color gradient at the bottom right. **(B)** The cooperation network map of institutions involved in DDH research (generated by CiteSpace). The nodes with a high BC-value (≥0.1) are indicated by purple rings.

The incorporated articles on DDH were contributed by at least 79 different countries and regions. The USA was the foremost productive country, with 657 articles published (24.4%), followed by China (376, 14%), Japan (323, 12%), and UK (316, 11.7%). As mentioned in Table 1, H-index in the USA 57 exceeded other countries, ranking first. The UK ranked second 38 and followed by Japan 35. The geographical distribution map of global publication revealed that articles on DDH had been published in various areas of the world except for some regions of Africa, Southeast Asia, and South America (Figure 3B). A transformative trend in the annual publication numbers of the top 10 countries from 1998 to 2020 was illustrated in Figure 3D. As shown in Figure 3C, international collaboration analysis indicated that the USA collaborated most closely with Canada, China, Japan, and the UK. The co-authorship network between countries was analyzed using VOSviewer software (Figure 4A). Only countries and regions with a minimum of five publications were included. Of the 47 countries and regions that met this threshold, the top five with the largest TLS were listed as follows: USA, UK, Canada, China, and Switzerland.

**Table 1:** Top 10 countries and institutions contributed to research publications in the developmental dysplasia of the hip (DDH) field.

**Rank**	**Countries**	**Counts**	**H-index**	**ACI**	**Institutions**	**Countries**	**Counts**	**H-index**	**ACI**
1	USA	657	57	22.18	Shanghai Jiao Tong University	China	52	13	7.67
2	China	376	23	6.88	Children's Hospital of Philadelphia	USA	50	16	14.32
3	Japan	323	35	14.24	Shriners Hospital for Children	USA	40	16	24.35
4	UK	316	38	17.81	Washington University	USA	34	16	41.09
5	Turkey	259	23	7.98	Hospital for Sick Children	Canada	32	14	17.31
6	Canada	128	23	15.48	Kyushu University	Japan	29	13	19.31
7	Germany	100	23	15.40	Mayo Clinic	USA	28	16	27.25
8	France	86	21	15.05	University of Bern	Switzerland	28	18	81.21
9	Switzerland	81	29	40.89	Boston Children's Hospital	USA	27	14	28.63
10	Netherlands	69	20	15.64	China Medical University	China	27	8	8

*ACI, average citation per item. Publications from Taiwan, Hong Kong, and Macau were assigned to China, and those from England, Northern Ireland, Scotland, and Wales were reclassified to the UK*.

### Contributions of Institutions

In terms of research institutions, only the top 10 are specifically laid out in Table 1. Of these, there were five American institutions, two Chinese institutions, and the other three came from Canada, Japan, and Switzerland, respectively. Among them, Shanghai Jiao Tong University had the largest number of publications 52 papers, followed by Children's Hospital of Philadelphia 50 papers, and Shriners Hospital for Children 40 papers. As for other indicators, the University of Bern had the highest value of H-index 18 and the most average number of citations (81.21). A cooperation visualization map of the DDH research network was generated by CiteSpace and illustrated in Figure 4B. The interinstitutional collaboration was relatively low and mainly conducted in European and American institutions. Children's Hospital of Philadelphia occupied the center location of the collaboration network and was the only institution with the BC-value >0.1.

### Contributions of Authors

**Figure 5:**
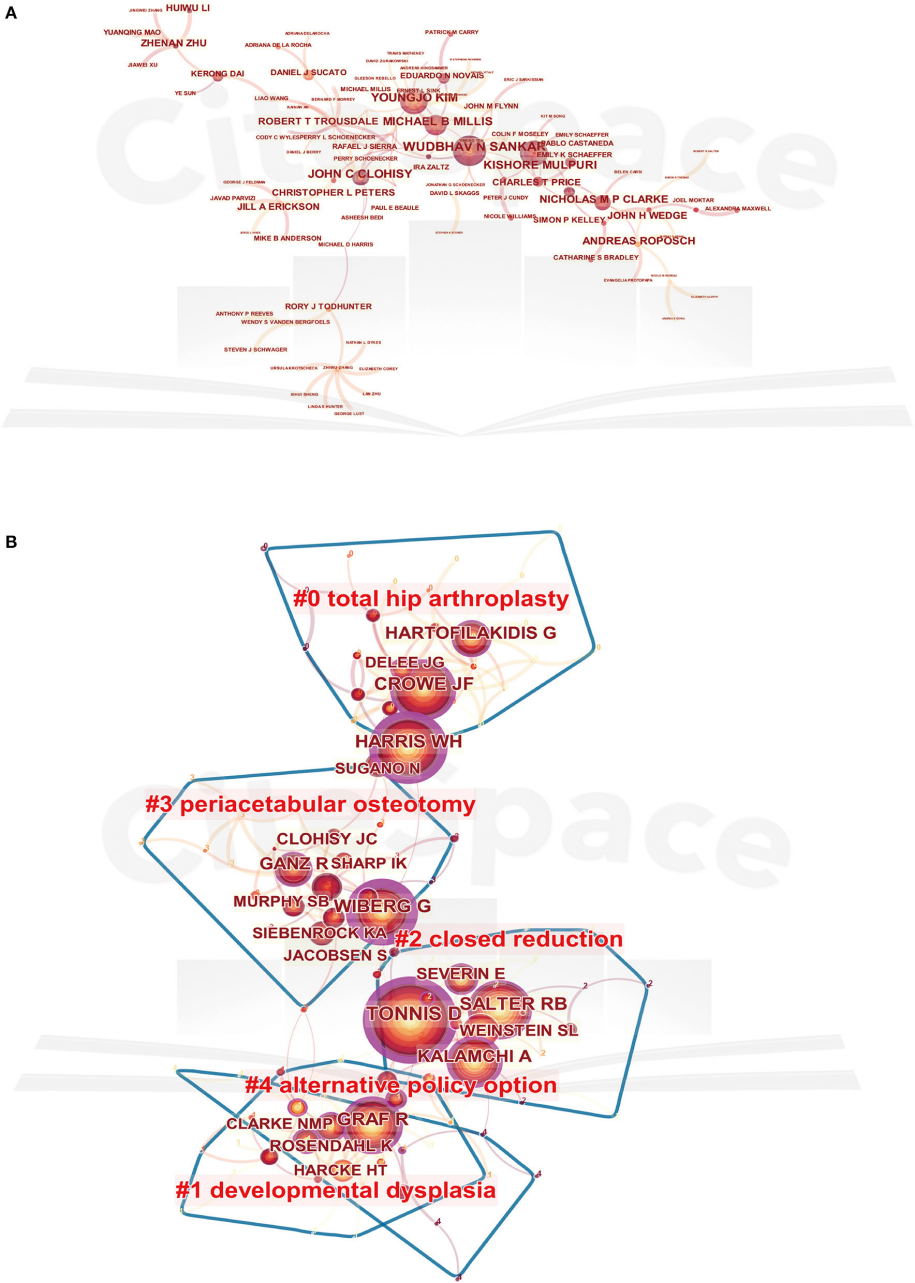
The network map of authors cooperation **(A)** and the cluster view map of co-cited authors **(B)**. In the cluster map, cited authors with similar categories were gathered in a cluster. All the clusters were labeled in different colors, and the links between nodes represented elements cited together. Both of the maps were generated by CiteSpace.

Table 2 lists the top 10 authors who published the greatest number of articles. Sankar WN from Children's Hospital of Philadelphia was the author with the most publications of 35, followed by Kim YJ and Clarke NMP. The CiteSpace software was used to analyze the cooperation and co-citation relationships between authors *via* creating network visualization maps. As shown in Figure 5A, the network map of author cooperation was a low-density map. The BC-values in all these authors were <0.1. As for the cluster view of the co-citation map (Figure 5B), the silhouette value of clusters #0 to #4 was from 0.892 to 0.995, showing good homogeneity. Research categories of authors were divided into five clusters, including “total hip arthroplasty,” “developmental dysplasia,” “closed reduction,” “periacetabular osteotomy,” and “alternative policy option.” And Tönnis Dwith, 650 co-citations, ranked first among the top 10 co-cited authors, followed by Harris WH, Crowe JF, Graf R, and Salter RB (Table 2).

**Table 2:** The top 10 most productive and co-cited authors in DDH research.

**Rank**	**Author**	**Counts**	**H-index**	**ACI**	**Co-cited author**	**Citation counts**	**Centrality**
1	Sankar WN	35	14	11.6	Tönnis D	650	0.64
2	Kim YJ	31	17	41.74	Harris WH	414	0.63
3	Clarke NMP	29	15	23.48	Crowe JF	410	0.22
4	Nakashima Y	27	13	18.81	Graf R	382	0.38
5	Clohisy JC	25	14	43.28	Salter RB	380	0.11
6	Millis MB	25	14	24.24	Wiberg G	373	0.91
7	Mulpuri K	22	8	10.27	Kalamchi A	307	0.24
8	Paton RW	22	11	16.59	Hartofilakidis G	299	0.11
9	Bicimoglu A	21	8	11.1	Ganz R	286	0.18
10	Roposch A	21	10	13.24	Weinstein SL	238	0.03

*ACI, average citation per item*.

### Analysis of Funding Agencies

**Figure 6:**
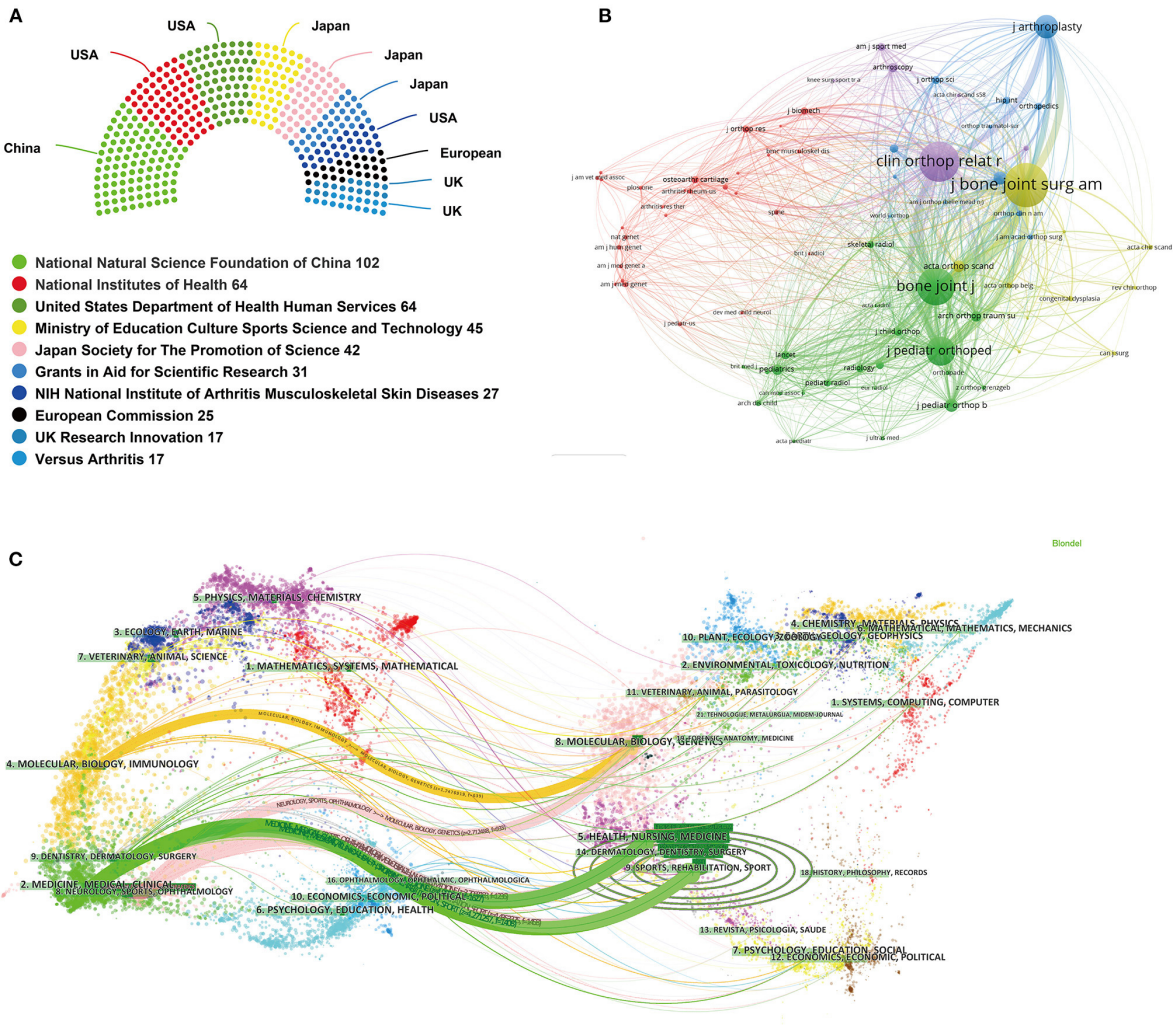
**(A)** Top 10 related funding agencies for the support of DDH research. **(B)** Journal co-citation analysis by using VOSviewer. Each node represents a different journal, and the node size is proportionate to the number of citations. **(C)** A dual-map overlay of the journals on DDH research by using CiteSpace.

Figure 6A displayed the top 10 related funding agencies for the support of DDH research. In terms of geographical distribution, three of them were from the USA, three from Japan, two from the UK, and one each from European Union and China. Specifically, the National Natural Science Foundation of China (NSFC) has sponsored the highest number of studies. National Institutes of Health (NIH) and United States Department of Health Human Services (HHS) ranked in the second and third places, respectively.

### Analysis of Core Journals and Subject Categories

Top 10 active journals published 1,289 articles on DDH, accounting for 47.9% of all 2,691 publications. The top 10 journals ordered by the number of publications are presented in Table 3. *Journal of Pediatric Orthopaedics* published the most articles/reviews (219 papers), accounting for 8.1% of the publications. *Journal of Bone and Joint Surgery-American Volume* has the largest impact factor of 4.578, and the highest value of H-index. According to the JCR 2019 standards, the top 10 most active journals were classified as Q1 in 4, Q2 in 3, Q3 in 2, and Q4 in 2. VOSviewer software was used to analyze the co-citation of journals. As shown in Figure 6B, 78 journals with at least 100 citations were included. The top three journals with the largest TLS were listed as follows: *Journal of Bone and Joint Surgery-American Volume, Clinical Orthopaedics and Related Research*, and *Bone Joint Journal*. A dual-map overlay of the journals on DDH research is shown in Figure 6C. As can be seen from this figure, there were five main citation paths in the dual-map including one orange path, two green paths and two pink paths. In addition, we also analyzed the co-occurring network of subject categories on DDH research by CiteSpace. As shown in Supplementary Figure 2, orthopedics, surgery, and pediatrics were the top three subject categories that received the most attention.

**Table 3:** Top 10 journals in the field of DDH research ranked by the publication number.

**Rank**	**Journal Title**	**Count**	**Percentage (N/2691)**	**IF (2019)**	**JCR (2019)**	**H-index**	**ACI**
1	*Journal of Pediatric Orthopaedics*	219	8.14	1.909	Q2/Q3	29	14.44
2	*Journal of Pediatric Orthopaedics Part B*	164	6.09	0.832	Q4	20	8.78
3	*Clinical Orthopaedics and Related Research*	163	6.06	4.329	Q1	36	36.72
4	*Journal of Arthroplasty*	163	6.06	3.709	Q1	30	19.32
5	*Bone Joint Journal*	157	5.83	4.306	Q1	34	24.17
6	*Journal of Bone and Joint Surgery American Volume*	113	4.20	4.578	Q2	41	39.47
7	*Hip International*	107	3.98	1.349	Q3	12	4.71
8	*International Orthopaedics*	96	3.57	2.854	Q1	18	11.65
9	*Archives Of Orthopaedic and Trauma Surgery*	54	2.01	2.021	Q2	14	10.06
10	*Journal of ChildrensOrthopaedics*	53	1.97	1.075	Q4	9	4.38

*ACI, average citation per item*.

### Keyword Analysis of Research Hotspots

**Figure 7:**
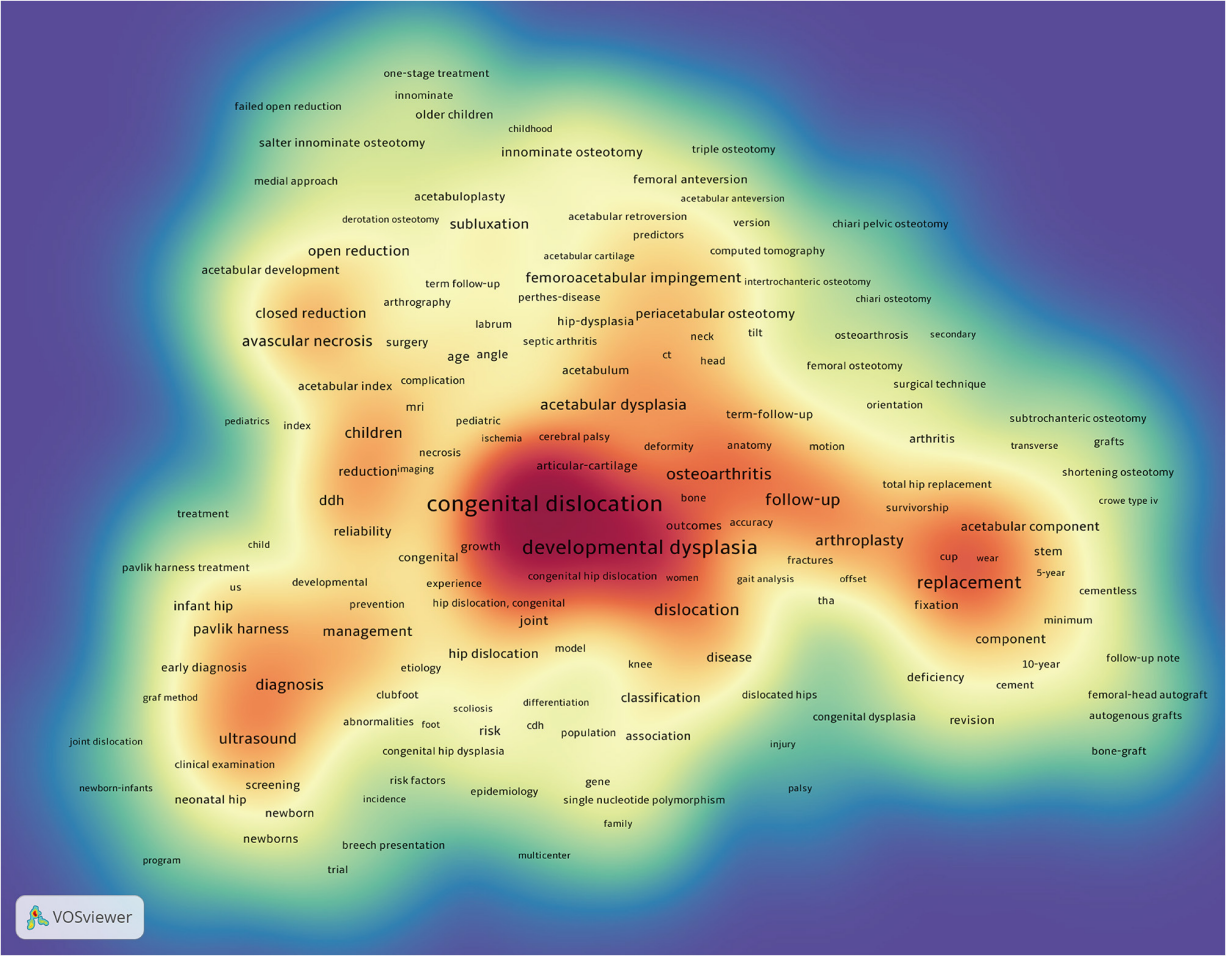
The density visualization map of keyword co-occurrence analysis (generated by VOSviewer). The darker the color, the higher the keywords density.

**Figure 8:**
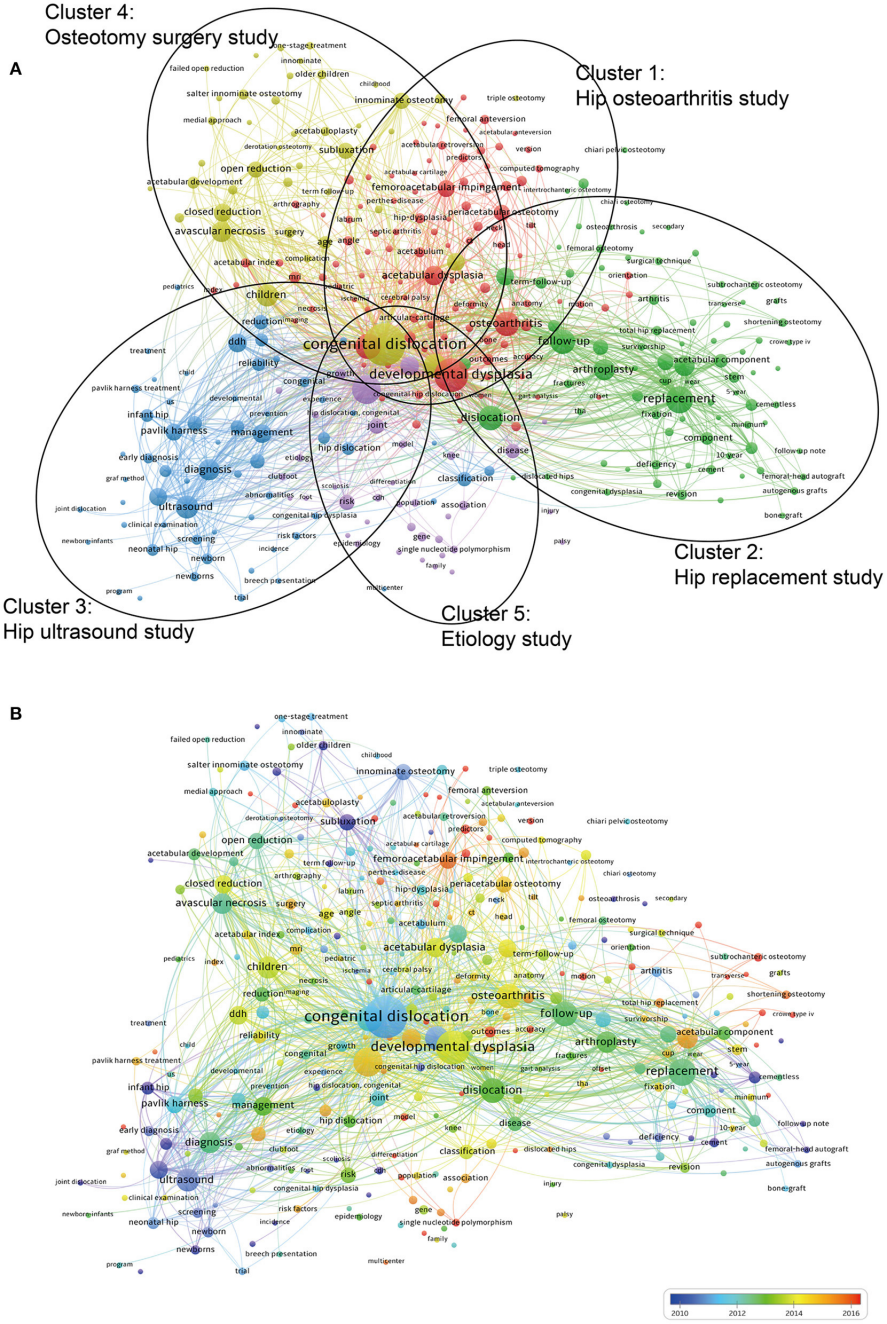
Keyword analysis of research hotspots. Network visualization **(A)** and the overlay visualization map **(B)** of the keywords co-occurrence analysis using VOSviewer.

A total of 5,663 keywords were extracted from 2,691 publications and analyzed by VOSviewer. As illustrated in the density visualization map of Figure 7, 324 keywords with the minimum number of occurrences over 10 times were included, and several hotspot clusters related to congenital dislocation, ultrasound, osteoarthritis, and replacement were observed. We further performed clustering analysis of these co-occurrence keywords, all of them could be classified into five clusters in Figure 8A: Cluster 1 (hip osteoarthritis study, red nodes); Cluster 2 (hip replacement study, green nodes); Cluster 3 (hip ultrasound study, blue nodes); Cluster 4 (osteotomy surgery study, yellow nodes); Cluster 5 (etiology study, purple nodes). These clusters showed the most prominent topics in DDH research so far. As shown in Figure 8B, different colors were applied for each keyword according to their appearance time in literature. The blue nodes represent the keywords that appear earlier, whereas the red nodes stand for the recent occurrence.

**Figure 9:**
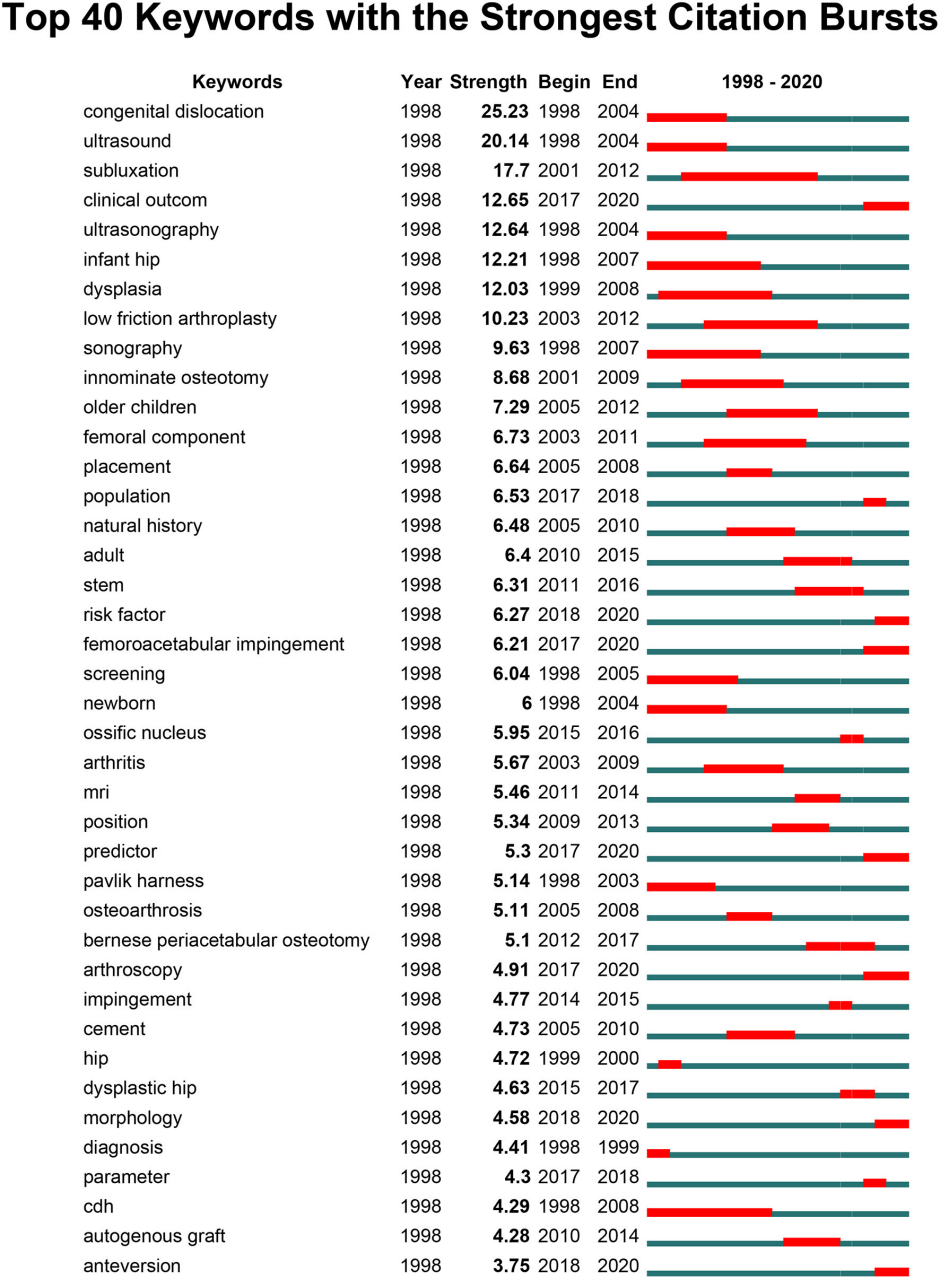
The top 40 keywords with the strongest citation bursts from 1998 to 2020. The blue line indicates the time interval, while the red line represents the duration in which a keyword was detected the strongest citation burst.

In addition, keyword co-occurring analysis was also performed by CiteSpace software (Supplementary Figure 3). The burst keyword by CiteSpace was considered another important indicator to reflect the research frontiers and emerging trends of a specific field. Keywords with the strongest citation bursts usually refer to these keywords being heavily cited by articles and acquiring tremendous attention over a period of time. The top 40 keywords with the strongest citation bursts from 1998 to 2020 are presented in Figure 9. Notably, the keywords with citation bursts that continue to 2020 are as follows: “clinical outcome (2017–2020),” “risk factor (2018–2020),” “femoroacetabular impingement (2017–2020),” “predictor (2017–2020),” “arthroscopy (2017–2020),” “morphology (2018–2020),” and “anteversion (2018–2020).”

## Discussion

In this study, we focused on the global trends in DDH research from aspects of primary publications, contributing countries, institutions, funding agencies, journals, and authors through bibliometric and visualized analysis. Over the last two decades, DDH has been actively researched, and the number of publications has steadily increased year by year. A total of 2,691 articles were searched using WoSCC. The number of publications has experienced rapid growth recently, which may indicate that more publications on DDH may become available in the coming years as a result of growing concern. According to the available knowledge, many factors contribute to the rapid progression of DDH research. One of the important reasons may be the popularization of the ultrasound screening of DDH, especially in some European and American countries, and the spread of the method over developing countries (27, 28).

As for the contributions of countries, the USA, China, Japan, and the UK have played important roles in DDH research, and their total numbers of publications occupied the top four, respectively. In the initial stages of research, the USA occupied the absolute dominant position due to superior conditions of basic research and clinical trials, including advanced techniques and equipment, professional researchers, as well as adequate funding. Nevertheless, this gap gradually decreased over the years as the growing interest of Asian countries and European countries in this area and an increasing number of related studies dominated by these countries. The increasing trend of these countries appears to be related to changes in their gross domestic product (29). Additionally, the H-index could also reflect the academic impact of one country (30). Consistent with the findings reported above, the USA far exceeded those in the other countries in terms of the H-index, making the largest contribution to global DDH research.

In terms of research institutions, all the top 10 institutions were located in the top 10 countries, and half of these institutions were located in the USA, which implied that establishing first-class research institutions are fundamental to advance up the academic ladder of a country. Meanwhile, although Shanghai Jiao Tong University had the largest number of publications, the value of the H index and ACI is much lower than other European and American institutions. Therefore, apart from the pursuit of the number of publications, attention should be paid to improve the quality of research articles. Furthermore, as can be seen from the cooperation visualization map, the interinstitutional collaboration was relatively low and mainly conducted in European and American institutions. Children's Hospital of Philadelphia was the only institution with a BC-value >0.1. Inadequate international collaboration may greatly reduce the efficiency and flexibility of the research. Collaboration of authors and more interinstitutional communication are also likely to improve the quality of research. Moving forward, collaborations and knowledge communication between institutions will continue to be emphasized.

For journals analysis, the *Journal of Pediatric Orthopaedics, Journal of Pediatric Orthopaedics Part B, Clinical Orthopaedics and Related Research, Journal of Arthroplasty*, and *Bone Joint Journal* have published the most articles on DDH research, ahead of other journals. Of these, *Journal of Pediatric Orthopaedics* and *Journal of Pediatric Orthopaedics Part B* focused on the studies of childhood orthopedic problems, while the other three were primary journals containing adult orthopedics publications. Consequently, it can be speculated that future developments in this field are more likely to be published in the listed journals. As for the dual-map overlay of the journals on DDH research, it can be seen that all the published studies mainly targeted journals in three fields: (i) medicine, medical, and clinical; (ii) sports; (iii) molecular, biology, and immunity. Whereas, the most cited publications originated from the journals of (i) sports, rehabilitation, and sport; (ii) health, nursing, and medicine; (iii) molecular, biology, and genetics.

Cooperation and co-citation analysis could reveal the collaboration network of the core authors and evaluate the academic influence of these authors in the DDH research field. As the core research strength in the field, their research findings may serve as an important reference for future studies. Authors such as Sankar WN, Kim YJ, and Clarke NMP published the largest number of papers in this field. They and their institutions exert important influence in the research area of emerging development in DDH. Moreover, Tönnis D, Harris WH, Crowe JF, and Graf R were rated as highly influential authors with a centrality of more than 0.1. The chief contribution of Tönnis D was proposing a modified technique of the triple pelvic osteotomy technique (31, 32). Unlike other peri-acetabular osteotomies, the key difference of the Tönnis method was the additional ischial osteotomy, which allowed satisfactory rotation of the acetabulum. Professor Harris WH is one of the top experts in the field of arthroplasty surgery from Massachusetts General Hospital. He has done a great deal of basic and clinical research and accumulated many scientific data on arthroplasty (33). The scoring criteria of Harris Hip Score (HHS) proposed by him have been widely utilized in Orthopedics and cited over 4,000 times (34). The major achievement of Professor Crowe JF was proposing a relatively simple classification system for dysplastic hips in adult patients, which has remained widely used by clinicians until today (35, 36). While the main contribution of professor Graf R was that he has proposed a novel method of ultrasound screening for DDH, and it has become the preferred method to evaluate acetabular development (37, 38). Furthermore, in the clustering analysis, “total hip arthroplasty,” “developmental dysplasia,” “closed reduction,” “periacetabular osteotomy,” and “alternative policy option” contained the largest authors group, which illustrated these research directions received the most attention.

In the present study, a total of 5,663 keywords were extracted from all the publications and analyzed by VOSviewer. Eventually, five keyword clusters were identified as the main research topics in this domain.

(i) Etiology study: To date, the pathogenic mechanism of DDH is complicated, and the exact etiology remains unclear. It is generally considered a multifactorial disease result from the interaction of genetic and environmental factors. Current known risk factors include breech presentation (39), oligohydramnios (40), positive family history (41), firstborn (41), and female sex (42). Newborn individuals with the above risk factors have a high prevalence of DDH. Therefore, identification of these risk factors is of great value for early identification and subsequent interventions. Research on the etiology of DDH, especially genetics of populations, has great potential to guide the prevention and treatment of DDH in the future.

(ii) Hip ultrasound study: Currently, the American Academy of Pediatrics has recommended that infants with risk factors of DDH are the relative indications for referral to an orthopedist (43). Traditionally, the radiological examination has been applied for the diagnosis of DDH, but, in the past two decades, ultrasound has been the gold standard for detecting DDH in children younger than 6 months, with a sensitivity of 100% (6, 14). Previous studies have demonstrated that using ultrasound was able to detect more cases, resulting in more children being treated earlier (14). So far, many ultrasound screening techniques have been applied to diagnose DDH, including Graf's technique (37, 38), Harcke's technique (44), Novick's technique (45), and so on. Among the mentioned techniques, Graf's was the most widely used and the preferred method (37, 38). But all the methods possess certain advantages and limitations. In addition, the necessity of widespread screening has been questioned, and many developing countries have yet to establish well-organized ultrasound screening guidelines. Therefore, further work still needs to be carried out regarding these aspects of etiology and ultrasound study of DDH.

(iii) Osteotomy surgery study: Generally speaking, children aged ranging between 1.5 and 8 years old or those who fail to restore a normal concentric structure of hip with closed reduction are considered candidates for osteotomy surgery. Osteotomy surgeries include femoral shortening osteotomy, pelvic osteotomy, and periacetabular osteotomy, and patients were treated with different surgical protocols based on age and imaging results (46).

(iv, v) Hip osteoarthritis and hip replacement study: Studies demonstrated that even after undergoing osteotomy surgeries, there is still a percentage of patients who progress to osteoarthritis and require arthroplasty (47). If left untreated, patients with early-stage DDH definitively develop secondary osteoarthritis, with 50% of these patients developing advanced osteoarthritis at the age of 50 years (48). Developmental dysplasia of the hip was recognized as a significant risk factor in hip osteoarthritis in adults. In China, 20–30% of patients with severe hip osteoarthritis are secondary to DDH and ultimately require THA surgery (49). Moreover, altered anatomy of the hip results in increased surgical complexity and risk at the time of arthroplasty. A previous study also found that patients with DDH undergoing primary THA incurred higher hospital costs than patients with primary osteoarthritis, which was mainly associated with the rising specialized implants costs (5, 10). However, in recent years, the continuous development of new surgical methods, new implants, new techniques, and devices, including virtual surgical planning, 3D virtual simulation system, navigation, and robot-assisted surgery, and the invention of more accurate imaging examination and diagnostic techniques all may contribute to bettering patient outcomes after THA surgery (50, 51).

As shown in Figure 8B, all the nodes were noted with different colors based on the average time of appearance. From the results, a trend of balanced development existed in the clusters of “hip replacement study,” “osteotomy surgery study,” and “hip ultrasound study” over the last two decades. In contrast, the clusters of “hip osteoarthritis study” and “etiology study” have attracted increasing attention since 2013. However, recent research trends have indicated that the other three clusters were also undergoing different degrees of developmental changes on the DDH research hotspots, which implied a diversified developing trend. Besides this, the temporal trend of research hotspot shifts based on the top 40 keywords with the strongest citation bursts from 1998 to 2020 was also analyzed by CiteSpace. Studies from 1998 to 2005 mainly focused on ultrasound, ultrasonography, infant hip, sonography, diagnosis, screening, and Pavlik harness, indicating that the researchers paid more attention to the early detection of DDH. From 2006 to 2012, the representative keywords were innominate osteotomy, low-friction arthroplasty, arthritis, placement, and nature history, which reflected the development of diverse therapeutic methods. While over the recent 4 years, from 2017 to 2020, the most frequently encountered keywords were “clinical outcome” (34–36), “risk factor” (52), “femoroacetabular impingement” (53), “predictor” (54), “arthroscopy” (55), “morphology” (56), and “anteversion” (57) and may continue to be the research hotspots in the near future.

## Limitations

Despite our comprehensive analysis of DDH research in this study, there were several limitations that need to be acknowledged. First, one of the limitations of this bibliometric analysis was the potential for incomplete retrieval of studies due to the restriction of the search terms. Nevertheless, we believed that the vast majority of DDH studies have been included, and the sample size was adequate. Second, all the bibliometric data included in this analysis were derived from a single WOSCC database. Thus, some of the relevant literature contained in other databases, such as Scopus, Embase, and PubMed, may have been missed inevitably. However, as mentioned in earlier studies, different databases employed counting methods of citations or different text export formats (26, 51). In this case, it may not be appropriate to combine data directly from different databases. Therefore, most of the previous bibliometric studies were conducted only based on one single database (24–26). Among them, the WOSCC database is one of the most popular public databases for bibliometric studies. Third, the inclusion of only English-based publications due to software limitations is another shortcoming, that several high-quality studies reported in non-English language were omitted.

## Conclusion

We summarized the publication information of DDH in the past 23 years, from 1998 to 2020, including contributions of countries, institutions, authors, funding agencies, and journals, and then analyzed the overall knowledge structure, development trends, and research hotspots in this field. It can be predicted that there will be an increasing number of publications on DDH research, and the United States stay ahead in this field. The global distribution of pieces of DDH research is imbalanced, and collaboration among institutions and authors needs to be strengthened. Furthermore, it is recommended to pay more attention to the following research topics: clinical outcome, risk factor, femoroacetabular impingement, predictor, arthroscopy, morphology, and anteversion.

## Data Availability Statement

The original contributions presented in the study are included in the article/Supplementary Material, further inquiries can be directed to the corresponding author/s.

## Author Contributions

HW, HY, and ZS designed the study. LT and YW collected the data. HW, YW, LT, HY, and ZS analyzed the data and drafted the manuscript. HY and ZS revised and approved the final version of the manuscript. All authors contributed to the article and approved the submitted version.

## Funding

This study was supported by the Tianjin Municipal Health Bureau (Grant No. 14KG115) and the Key projects of Tianjin Natural Science Foundation (Grant No. 20JCZDJC00730).

## Conflict of Interest

The authors declare that the research was conducted in the absence of any commercial or financial relationships that could be construed as a potential conflict of interest.

## Publisher's Note

All claims expressed in this article are solely those of the authors and do not necessarily represent those of their affiliated organizations, or those of the publisher, the editors and the reviewers. Any product that may be evaluated in this article, or claim that may be made by its manufacturer, is not guaranteed or endorsed by the publisher.

## Abbreviations

DDH: developmental dysplasia of the hip
THA: total hip arthroplasty
WOS: web of science
TLS: total link strength
HHS: Harris hip score
ACI: average citation per item
BC: betweenness centrality.
