# Supplementary material for: The Global Research Trends and Hotspots on Developmental Dysplasia of the Hip: A Bibliometric and Visualized Study
Source: Front Surg. 2021 Oct 25;8:671403. doi: 10.3389/fsurg.2021.671403 (PMC8572967; doi:10.3389/fsurg.2021.671403)
Supplement: Supplementary file 1 [file Data_Sheet_1.DOCX]

Supplementary Material

# Supplementary Figures and Tables

## Supplementary Figure


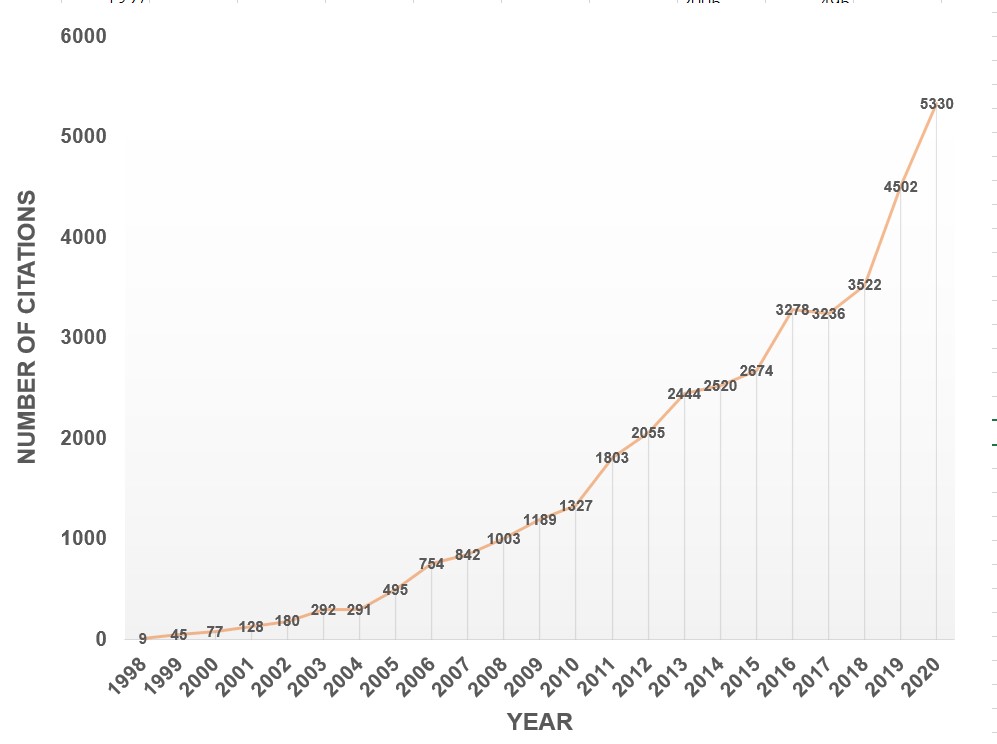


**Supplementary Figure 1.** The summed total citations of annual articles related to DDH.


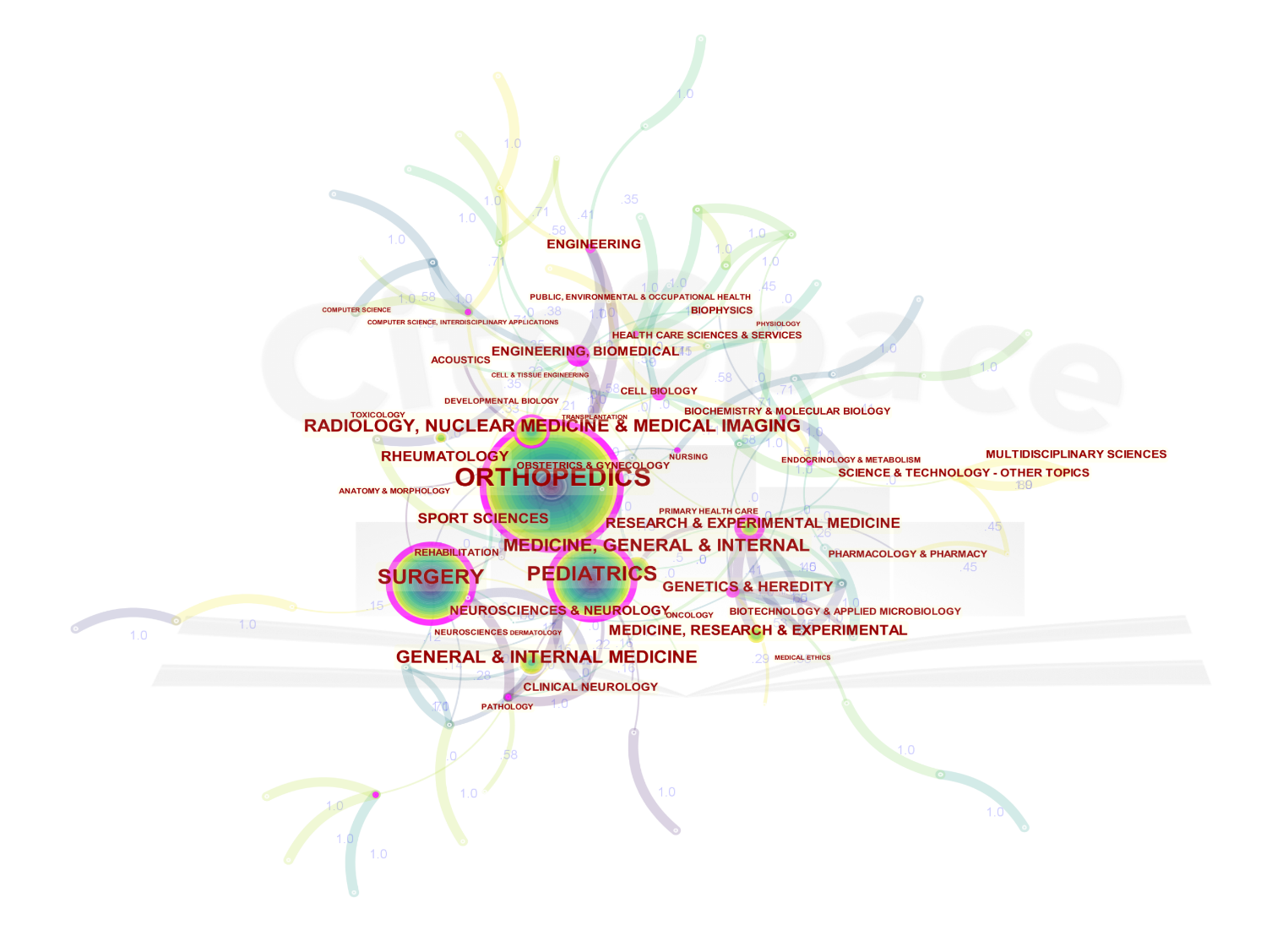


**Supplementary Figure 2.** Co-occurring network map of subject categories on DDH research generated by CiteSpace.


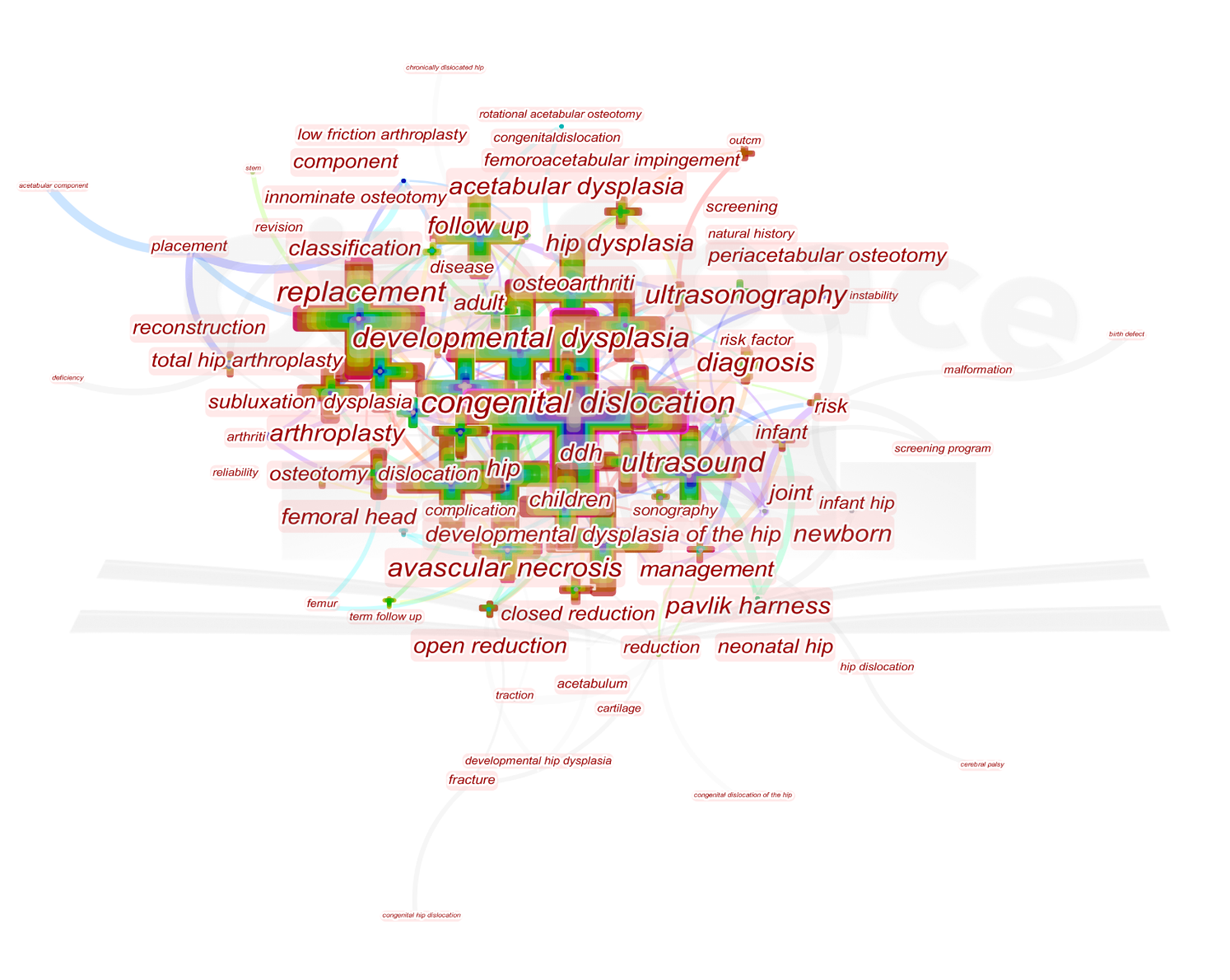


**Supplementary Figure 3.** Co-occurring network map of keywords on DDH research generated by CiteSpace.
